# Spatial and Temporal Trends in Insecticide Resistance among Malaria Vectors in Chad Highlight the Importance of Continual Monitoring

**DOI:** 10.1371/journal.pone.0155746

**Published:** 2016-05-26

**Authors:** Geraldine Marie Foster, Michael Coleman, Edward Thomsen, Hilary Ranson, Elise Yangalbé-Kalnone, Tchomfienet Moundai, Israel Demba Kodindo, Amen Nakebang, Adoum Mahamat, Mallaye Peka, Clement Kerah-Hinzoumbé

**Affiliations:** 1 Department of Vector Biology, Liverpool School of Tropical Medicine, Liverpool, United Kingdom; 2 Programme National de Lutte contre le Paludisme, N’Djamena, Chad; 3 Programme National de Lutte contre l’Onchocercose, Ministère de la Santé Publique, N’Djamena, Chad; 4 Programme National de Lutte contre la Trypanosomiase Humaine Africaine, Ministère de la Santé Publique, N’Djamena, Chad; Kansas State University, UNITED STATES

## Abstract

**Background:**

A longitudinal *Anopheles gambiae* s.l. insecticide resistance monitoring programme was established in four sentinel sites in Chad 2008–2010. When this programme ended, only sporadic bioassays were performed in a small number of sites.

**Methods:**

WHO diagnostic dose assays were used to measure the prevalence of insecticide resistance to 0.1% bendiocarb, 4% DDT, 0.05% deltamethrin, 1% fenitrothion, and 0.75% permethrin in the main malaria vectors at the beginning and end of the malaria transmission season for three years 2008–2010, with subsequent collections in 2011 and 2014. Species and molecular identification of *An*. *gambiae* M and S forms and *kdr* genotyping was performed using PCR-RLFP; circumsporozoite status was assessed using ELISA.

**Results:**

Between 2008 and 2010, significant changes in insecticide resistance profiles to deltamethrin and permethrin were seen in 2 of the sites. No significant changes were seen in resistance to DDT in any site during the study period. Testing performed after the period of routine monitoring had ended showed dramatic increases to DDT and pyrethroid resistance in 3 sites. No resistance to organophosphate or carbamate insecticides was detected. *An*. *arabiensis* was the predominate member of the *An*. *gambiae* complex in all 4 sites; adult collections showed temporal variation in species composition in only 1 site. *Kdr* analysis identified both 1014F and 1014S alleles in *An*. *gambiae* S only. Circumsporozoite analysis showed the highest vector infection rates were present in Donia, a site with extensive use of agricultural insecticides.

**Conclusions:**

During the monitoring gap of four years, significant changes occurred in resistance prevalence in 3 of the 4 sites (p = <0.001), endangering the efficacy of currently implemented malaria control interventions. Significant changes in insecticide resistance profiles and a lack of *kdr* resistance alleles in adult populations highlight the urgent need for comprehensive entomological monitoring to be implemented and sustained in country.

## Introduction

Since 2000, the expansion of malaria control programmes has led to significant reductions in malaria morbidity and mortality [1]. Indoor residual spraying (IRS) and long lasting insecticide-treated bed nets (LLINS) are the most extensively implemented vector-reduction techniques, and the use of these interventions has led to a rapid scale-up in the use of insecticides as a tool for malaria control [1].

The pool of insecticides available for IRS and LLIN is critically limited. Twelve insecticides are currently approved by the World Health Organization (WHO) for IRS, belonging to just four chemical classes (organochlorines, organophosphates, carbamates and pyrethroids). For LLINs, the range of available insecticides is even more limited and currently only six pyrethroid insecticides are available (WHO pesticide evaluation scheme, http://www.who.int/whopes/en/).

Prolonged exposure to insecticides has inevitably led to the selection of insecticide resistance in the malaria vector, *Anopheles*, with the first reports of resistance to the organochlorine DDT in African malaria vectors emerging in the mid-20^th^ century [2,3]. Pyrethroid resistance reports followed in 1993 [4], and is now widespread across Africa (www.irmapper.com). Resistance to carbamates and organophosphates has been reported in Anopheline mosquitoes in several African countries and is spreading rapidly (www.irmapper.com). Given the limited range of insecticides available, understanding the patterns and extent of resistance is vital for effective malaria control.

The WHO/TDR network for insecticide resistance in African malaria vectors was established in 2008 with the objective of improving the monitoring of insecticide resistance in malaria vectors, particularly in areas with large scale insecticide-based control programmes implemented or planned in the absence of data on vector susceptibility [5]. The network covered five countries (Angola, Burkina Faso, Benin, Chad and Sudan) and gathered biannual data over three years [5].

In Chad, 616,722 malaria cases were reported in 2012, an increase of over 200,000 cases since 2006 [6]. Overall, 80% of the population in Chad is at high risk of malaria [1]. Malaria transmission is seasonal and the main vectors are *An*. *arabiensis* and *An*. *gambiae* s.s. Both the M (*An*. *coluzzii*) and S forms of *An*. *gambiae* are present; minor vectors include *An*. *funestus*, *An*. *pharoensis* and *An*. *ziemanni* [7,8].

Currently, the most widely used vector control strategy in Chad is insecticide treated nets (ITNs), while IRS is planned for use in epidemic prone areas of the northern part of the country. Few studies assessing Anopheline resistance to insecticides have been performed in Chad. A study performed in 2006 looked at three districts where ITNs were implemented and found evidence of increased tolerance to pyrethroid insecticides but full sensitivity to carbamates and organophosphates [9]. More recently, investigations into resistance mechanisms in pyrethroid-resistant mosquitoes collected from the capital, N’Djamena, found that resistance was largely conferred by a single locus and that target site resistance was absent in this population [10]. As there is a relatively high level of gene flow between *An*. *arabiensis* populations, particularly in area with extensive use of LLIN [11], monitoring insecticide resistance in Chad is recommended to ensure the continuing efficacy of currently implemented control interventions.

This paper reports a comprehensive analysis of multi-year, multi-site entomological and insecticide resistance data from Chad.

## Materials and Methods

### Study area

**Fig 1:**
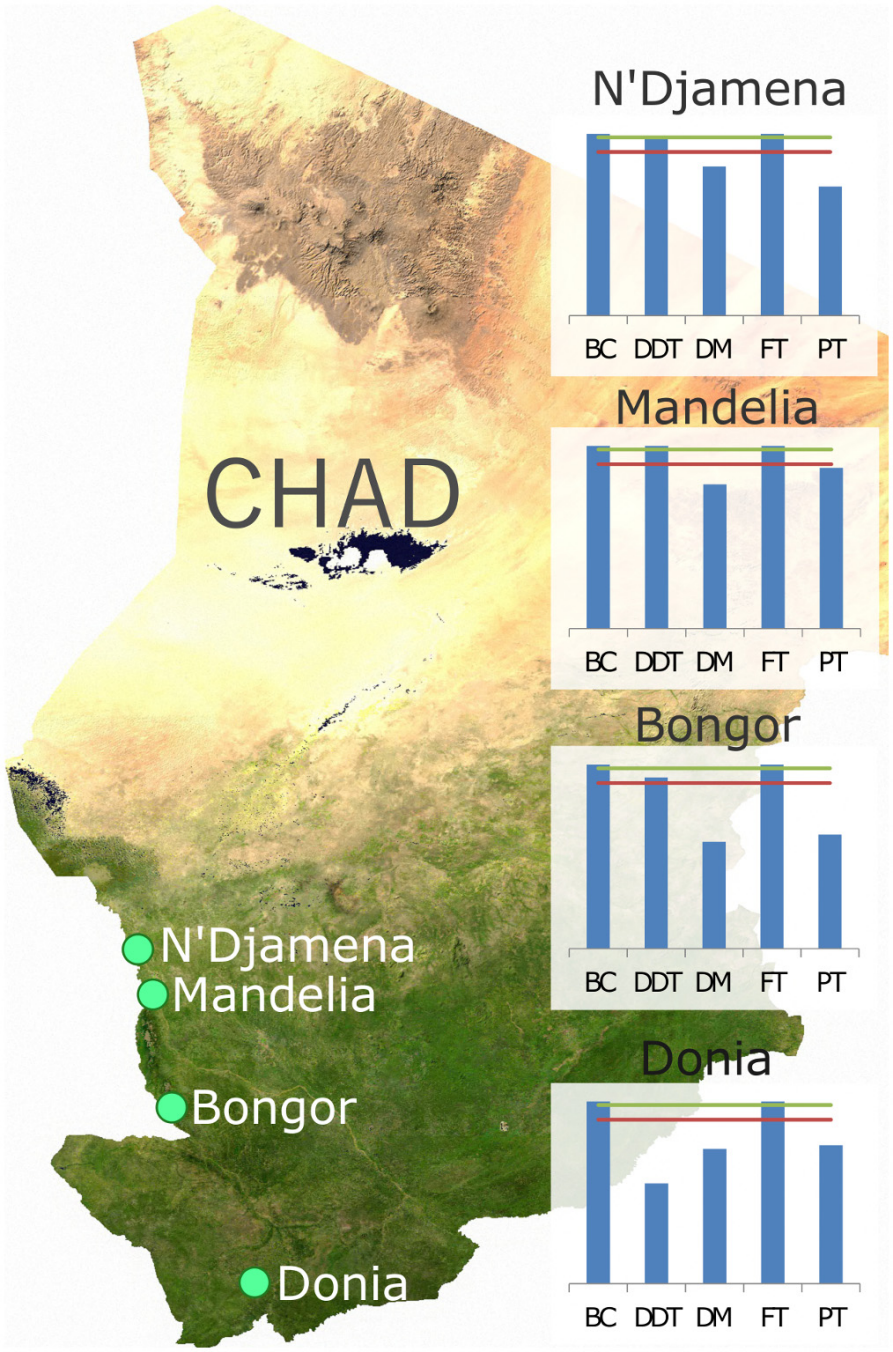
Sentinel sites for biannual resistance monitoring. Green dots indicate the geographic locations of each of the four sites. Sites were chosen to represent a range of different selection pressures for insecticide resistance. Embedded charts show the baseline percentage mortality for the year in which monitoring began (2008), where BC = bendiocarb, DDT = DDT, DM = deltamethrin, FT = Fenitrothion, and PT = Permethrin. Green lines represent 98% mortality and red lines indicate 90% mortality. Map source https://commons.wikimedia.org/wiki/File:Chad_sat.jpg.

This study was performed as part of a wider WHO/TDR project established to monitor insecticide resistance in five countries in Africa [5]. Four sentinel sites were established in Chad for biannual resistance monitoring (Fig 1). Each site was in a malaria endemic region and boundaries were defined by a 10km radius from a public healthcare facility. Sites were chosen to fulfil a range of insecticide selection pressure criteria and included an urban agricultural site with local use of insecticides (N'djamena, N 12° 6’, E 15° 02’), a rural site with intensive crop cultivation (Donia, N 8° 24’, E 16° 24’), a site with a high coverage of insecticide-based malaria control interventions (Bongor, N 10° 16’, E 15° 21’) and a site with no intensive agriculture and no organized vector control programme (Mandelia, N 11° 43’, E 15° 14’). The field sites were selected under the authority of the National Malaria Control Programme in Chad. No specific further permissions were required for the collection of either larval or adult mosquitoes from these sites and no endangered or protected species were involved.

### Mosquito collections

Sample collection times in each site were influenced by climate. In the semiarid sahelian zone (N’Djamena and Mandelia) there is a rainy season from June to September and a dry period from October to May. Sample collection times were July to early September. The humid soudanian zone (Bongor and Donia) has a rainy season between April and October. Sample collection dates here were late August to late October.

### Larval Collections and Insecticide Susceptibility Assays

Two rounds of mosquito collection were performed in July-August and September-October each year during which *Anopheles gambiae* s.l. were sampled as larvae from temporary water bodies encompassing the diversity of productive anopheline breeding sites within a 10km radius. Collections were performed over several days to minimize the probability of analysing siblings. All larval collections were pooled and reared to adult stage. Emerging mosquitoes were sexed, morphologically identified and non-blood fed, three to five day old females were used for susceptibility tests. Insecticide susceptibility tests were performed using WHO standard procedures and test kits for adult mosquitoes [12]. Briefly, a minimum of 100 mosquitoes were exposed to each insecticide for 1 hour, in batches of 20, during which the number of knocked down mosquitoes was recorded every five minutes. Following exposure, mosquitoes were transferred to holding tubes and supplied with a 10% sugar solution for 24h before scoring final mortality. A control tube with untreated papers was run simultaneously. Abbot's formula was used to correct mortality if necessary. Insecticides tested were: 0.1% bendiocarb, 4% DDT, 0.05% deltamethrin, 1% fenitrothion and 0.75% permethrin. Control mosquitoes as well as dead or alive specimens were individually stored in labelled tubes containing silica gel for molecular analysis. Quality control of each batch of WHO test papers was performed at the Liverpool School of Tropical Medicine using the *An*. *gambiae* Kisumu susceptible strain.

Tests for synergism with piperonyl butoxide (PBO) were conducted in conjunction with permethrin and deltamethrin. For each pyrethroid, 100 mosquitoes were exposed to 4% PBO for one hour then transferred immediately into tubes containing insecticide papers for a further one hour. Mortality was recorded after 24 hours in a same way as described above and compared to samples exposed directly to permethrin and deltamethrin papers. A PBO-only control was performed in conjunction with each test.

Following the six rounds of collections during 2008–2010, subsequent larval collections were performed in Donia (2011) and N’Djamena and Bongor (2014).

### Adult collections

Two rounds of collection of adult *Anopheles* mosquitoes were performed each year in each site between July 2008 and October 2010, timed to coincide with the peak of the malaria transmission season. Indoor collections were performed using pyrethrum spray catches (PSC) in 15 randomly selected houses and from window exit traps (ET) attached to nine randomly selected PSC houses. PSC were performed in all sites in each collection round and exit trap collections were performed in rounds 4, 5 and 6 only. In each selected house, one trap was installed overnight (ET1) and another in the morning after removal of the first trap (ET2). Outdoor collections were performed using pit traps in all six rounds of collection. Two pit traps were placed in each of the four sites. All specimens were classified according to their abdominal appearance and scored as unfed, blood-fed, semi-gravid or gravid and later preserved in labelled vials containing silica gel.

### Molecular identification, detection of *kdr* mutations and vector status determination

Sub-samples of 40 mosquitoes drawn from bioassay control mosquitoes were identified using PCR-RFLP [13] following DNA extraction [14]. Genotype at the *kdr* locus was determined using PCR assays described by Martinez-Torres et al [15] and Ranson et al [16]. Genotyping for the *ace*1^R^ mutations was performed according to the method in Weill et al [17].

Mosquitoes collected resting indoors, outdoors or exiting from houses were analysed for *Plasmodium falciparum* circumsporozoite status using enzyme-linked immuno-sorbent assays (ELISA) to determine vector status [18]. The sporozoite rate (SR) was calculated as the proportion of mosquitoes positive for *P*. *falciparum* circumsporozoite protein out of the total tested.

### Data analysis

Bioassay data were stratified by insecticide, location, collection round and year for analysis. Mortality rates for each insecticide were defined using WHO guidelines whereby resistant populations displayed less than 90% mortality, potentially resistant populations showed 90–98% mortality, and susceptible showed mortality greater than 98% [12]. Confidence limits (95%) were calculated using Microsoft Excel and bioassay mortalities were compared using Fisher’s exact test, using pooled data from both rounds of testing. The z-test was used to compare the proportions of molecular forms. Genotypic frequencies at the *kdr* locus were compared with Hardy-Weinberg proportions using the exact test procedures implemented in Genepop 3.4 software [19]. As mortalities in the control groups were less than 5%, no corrections using Abbot’s formula were required.

## Results

### Insecticide Bioassays

**Fig 2:**
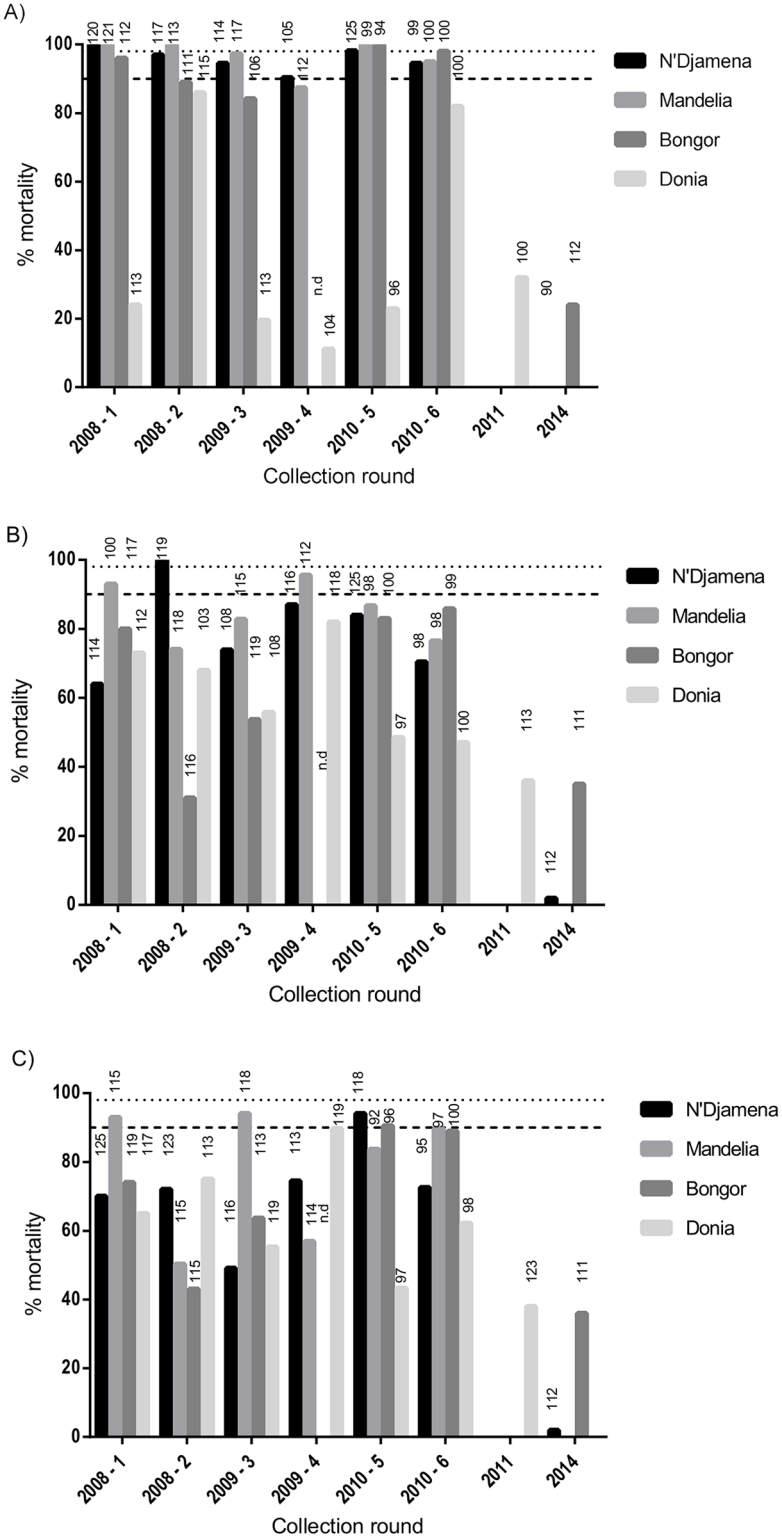
Results from insecticide resistance bioassays. A) DDT; B) Deltamethrin; C) Permethrin. No round 4 data from Bongor was available owing to the early end of the rainy season in 2009. Numbers in each column represent the number of mosquitoes exposed to insecticide paper. Data from 2008 have been published previously and are included for comparison.

Between 2008 and 2010, no resistance to bendiocarb or fenitrothion was detected in any sentinel site (data not shown). In N’Djamena, Mandelia and Bongor, populations were either fully susceptible to DDT or a low prevalence of resistance was observed (Fig 2a). Results from Donia showed resistance to DDT in every collection round. Despite the constant presence of resistance to DDT in Donia, no significant change over the study period was observed overall (p = 0.866, Table 1). A subsequent collection performed in Donia in 2011 showed that mortality to DDT had further declined to 32%. Additional collections in 2014 were performed in N’Djamena and Bongor, which showed a dramatic decrease in susceptibility to DDT to 0% and 24%, respectively (Fig 2a, Table 1, p = <0.001).

**Table 1:** Insecticide bioassay results for *Anopheles gambiae s*.*l*. in 2008, 2010 and 2014 in four sentinel sites in Chad.

Insecticide	Site	2008 mortality (95% CI)	2010 mortality (95% CI)	2014 mortality (95% CI)	p (2008 vs. 2010)	p (2010 vs. 2014)	p (2008 vs 2014)
Deltamethrin	Ndjamena	82 (70–94)	78 (66–90)	2 (0–5)	0.726	**<0.001**	**<0.001**
	Mandelia	79 (68–90)	82 (72–91)		0.883		
	Bongor	58 (39–77)	84 (78–100)	35 (33–37)	**0.013**	**<0.001**	**0.035**
	Donia	74 (69–12)	48 (40–56)		**0.006**		
Permethrin	Ndjamena	71 (63–79)	84 (70–98)	2 (0–5)	0.204	**<0.001**	**<0.001**
	Mandelia	88 (84–92)	86 (76–96)		1		
	Bongor	62 (51–73)	90 (86–94)	36 (32–40)	**0.015**	**<0.001**	**0.022**
	Donia	76 (70–82)	53 (40–67)		**0.024**		
DDT	Ndjamena	98 (96–100)	97 (93–100)	0 (-)	0.947	**<0.001**	**<0.001**
	Mandelia	100 (-)	98 (95–100)		0.891		
	Bongor	93 (88–98)	97 (94–100)	24 (21–27)	0.780	**<0.001**	**<0.001**
	Donia	55 (35–75)	53 (30–76)		0.866		

Data from biannual collections was pooled for analysis. P values were calculated using Fisher’s exact test, using absolute numbers of mosquitoes. Between 2008 and 2010 the prevalence of resistance in Bongor to deltamethrin and permethrin decreased, but this had rebounded by 2014.

No significant change in resistance to permethrin or deltamethrin was observed in N’Djamena and Mandelia during the 3 year study period (permethrin: p = 0.204 and p = 1, respectively; deltamethrin: p = 0.726 and p = 0.883, respectively; Table 1). However, collections in N’Djamena in 2014, four years after routine monitoring had ceased, showed a significant decrease in mortality to both insecticides (2%, p = <0.001; Table 1).

Significant changes to pyrethroid resistance were observed in Bongor and Donia during the study period (Table 1). In Bongor, there were significant increases in mortality to both deltamethrin and permethrin between 2008 and 2010 (p = 0.013 and p = 0.015, respectively; Table 1); in Donia, the opposite effect was observed, and a significant decrease in mortality to both pyrethroid insecticides was observed between 2008 and 2010 (p = 0.006 and p = 0.024; Table 1). The collection in Donia in 2011 following the end of the routine monitoring period showed a continuing decrease in mortality to deltamethrin and permethrin (Fig 2B, 36% and 38%, respectively), and collections made in Bongor in 2014 showed a significant decrease in mortality compared to 2010 and 2008 (Table 1).

Prior exposure of mosquito populations to PBO was performed in 2009 and 2010 and restored full susceptibility to permethrin and deltamethrin to populations from N’Djamena, Mandelia and Bongor (S1 Table). Populations from Donia could only be tested in one instance in 2009 using deltamethrin; this showed partial restoration of susceptibility (97.4% from 82.2%).

### Molecular Identification and kdr genotyping of control mosquitoes

Overall, 629 unexposed *An*. *gambiae* s.l. mosquitoes were identified to species level (S2 Table). In 2008 and 2010, *An*. *arabiensis* was the predominant species collected in N’Djamena, Mandelia and Bongor. The M form of *An*. *gambiae* s.s. was observed in low numbers in Bongor in 2008 and N’Djamena in 2010, however was found consistently in Donia throughout the collection period in 2008 and 2009. The S form of *An*. *gambiae* was only found in Donia. No significant difference was seen in the composition of mosquito populations between the start and the end of the transmission season, with the exception of Donia in 2009 (p = <0.001, Table 2).

**Table 2:** Molecular identification of unexposed *An*. *gambiae s*.*l*. mosquitoes collected in six rounds of collection between 2008 and 2010.

Year	Locality	Round 1 collections			Round 2 collections			Difference between start and end of season (p)
		Species	N	Proportion (%)	Species	N	Proportion (%)	
	N'Djaména	*An*. *arabiensis*	40	100.0	*An*. *arabiensis*	37	100.0	1
	Mandélia	*An*. *arabiensis*	39	100.0	*An*. *arabiensis*	39	100.0	1
		*An*. *arabiensis*	37	94.9	*An*. *arabiensis*	40	100.0	0.241
**2008**	Bongor	*An*. *gambiae M*	3	7.7				
		*An*. *arabiensis*	3	8.3	*An*. *arabiensis*	9	23.7	0.090
	Donia	*An*. *gambiae M*	14	38.9	*An*. *gambiae M*	17	44.7	
		*An*. *gambiae S*	19	52.8	*An*. *gambiae S*	12	31.6	
		*An arabiensis*	1	2.5	*An arabiensis*	27	67.5	<0.001
**2009**	Donia	*An gambiae M*	7	17.5	*An gambiae M*	7	17.5	
		*An gambiae S*	32	80.0	*An gambiae S*	6	15.0	
**2010**	N'Djamena	*An arabiensis*	40	100.0	*An*. *arabiensis*	39	97.5	1
					*An*. *gambiae M*	1	2.5	
	Mandelia	*An*. *arabiensis*	40	100.0	*An arabiensis*	40	100.0	1
	Bongor	*An*. *arabiensis*	40	100.0	*An arabiensis*	40	100.0	1

*An*. *arabiensis* was consistently the predominant species in N’Djamena, Mandelia and Bongor.

Genotyping of control mosquitoes collected in 2008 and 2010 showed no *An*. *arabiensis* or *An*. *gambiae* M with *kdr* mutations or the *Ace-1R* mutation in any site (n = 560, S2 Table). However, both 1014F and 1014S *kdr* mutations were found in Donia in *An*. *gambiae* S in 2008 (no molecular testing performed in 2009, and no collections performed in Donia in 2010). The frequencies of the two mutations were quite similar and were higher at the beginning [f(Ser) = f(Phe) = 0.37] than at the end [f(Ser) = f(Phe) = 0.13] of the rainy season (Table 3). All possible genotypes were observed at the *kdr* locus and in some cases, both mutations occurred in the same individual.

**Table 3:** *kdr* mutations frequencies among pools of unexposed An. gambiae M and An. gambiae S collected in 2008 and 2010 in three sites.

Collections	Localities	Species	N	Genotype at the *kdr* locus						f (Ser)	f (Phe)
				Leu-Leu	Leu-Phe	Phe-Phe	Leu-Ser	Ser-Ser	Phe-Ser		
	Bongor	*An*. *gambiae M*	3	3	0	0	0	0	0	0	0
**Round 1**	Donia	*An*. *gambiae M*	14	14	0	0	0	0	0	0	0
		*An*. *gambiae S*	19	1	4	3	4	3	4	0.37	0.37
**Round 2**	Donia	*An*. *gambiae M*	17	17	0	0	0	0	0	0	0
		*An*. *gambiae S*	12	6	3	0	3	3	3	0.13	0.13
**Round 6**	N'Djamena	*An*. *gambiae M*	1	1	0	0	0	0	0	0	0

### Adult collections

**Fig 3:**
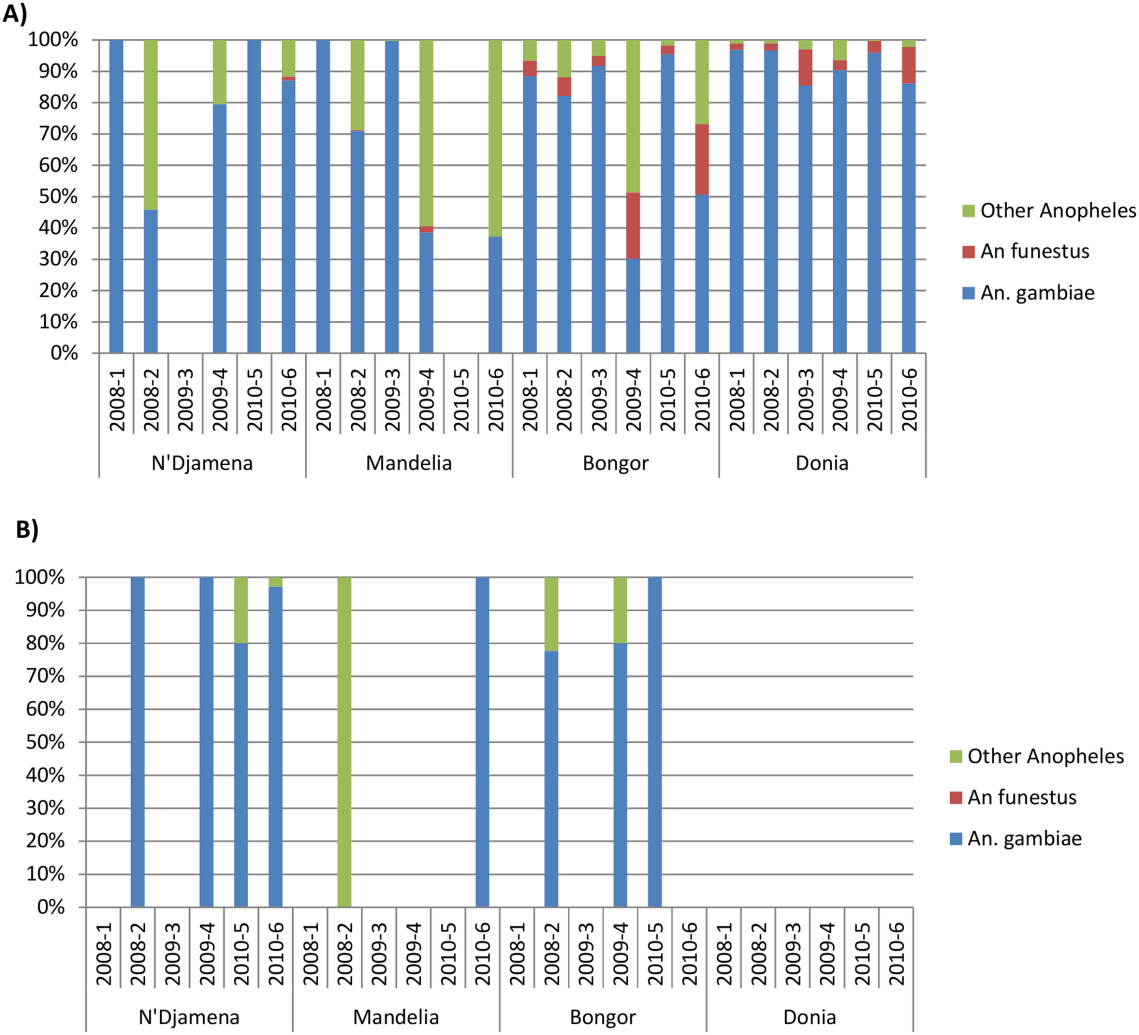
Results from adult collections. A) PSC B) Pit traps. Different coloured series represent different species: Green = Other Anopheles; Red = *An*. *funestus*; Blue = *An*. *gambiae*.

Overall, 7,308 anopheline mosquitoes were collected from PSC, Pit and Exit traps, comprising seven species (*An*. *gambiae* s.l., *An*. *funestus*, *An*. *pharoensis*, *An*. *rufipes*, *An*. *nili*, *An*. *squamosus*, *An ziemanni*). Of these, 5,168 mosquitoes were morphologically identified as *An*. *gambiae* s.l. (4,353 from PSC, 86 from Pit traps, 700 from ET1 and 29 from ET2, respectively) and 339 as *An*. *funestus* (292 from PSC, 0 from Pit traps, 36 from ET1 and 11 from ET2, respectively). *An*. *gambiae* s.l. was consistently the predominant species collected from PSC during the first collection round of each year in each site in N’Djamena, Mandelia and Bongor (Fig 3a); in Donia, the proportion of *An*. *gambiae* s.l. collected was consistent in each collection round. Few mosquitoes were collected from outdoor traps (Fig 3b). When mosquitoes were collected, *An*. *gambiae* s.l. was consistently the predominant species found in N’Djamena and Bongor, and the only one collected in Mandelia. No Anopheline mosquitoes were collected outdoors in Donia whatever the sampling period.

### Sporozoite analysis

Overall, 2,749 Anopheline mosquitoes collected in 2008 and 2010 were tested for *P*. *falciparum* circumsporozoite protein (Table 4). Infected *An*. *gambiae* s.l. were found in one collection round in Bongor in 2008 (SR = 0.5%) and in N’Djamena in 2010 (SR = 2.5%), however the highest infection rates were seen in Donia, where infected vectors were found in all four collection rounds (2008 SR = 7.5% and 15.5%; 2010 SR = 11.5% and 13.8%, respectively); no significant difference was seen between the numbers of infected vectors found at the start and end of the rainy season in either year (2008: p = 0.096; 2010 p = 0.69). Across all sites, there was no significant difference seen between the number of infected mosquitoes at the end of each rainy season (p = 0.80) or between the start and end of the rainy season in 2010 (p = 0.69); however, the number of infected mosquitoes at the end of the rainy season in 2008 was significantly higher than the number of infected mosquitoes at the start of the rainy season (p = 0.03).

**Table 4:** Circumsporozoite analysis from 2008 and 2010.

Year	Locality	CSP status	Round 1 collections		Round 2 collections	
			*An*. *gambiae s*.*l*.	*An*. *funestus*	*An*. *gambiae s*.*l*.	*An*. *funestus*
		N	21	0	11	0
	N’djamena	N+	0	-	0	-
		SI	0	-	0	-
		N	200	16	172	57
**2008**	Bongor	N+	1	1	0	0
		SI	0.5	6.2	0	0
		N	200	7	200	13
	Donia	N+	15	0	31	0
		SI	7.5	0	15.5	0
	Total infected mosquitoes		17		31	
		N	103	0	283	2
	N’Djamena	N+	0	-	7	0
		SI	0	-	2.5	0
		N	327	12	130	44
2010	Bongor	N+	0	0	0	0
		SI	0	0	0	0
		N	217	38	80	10
	Donia	N+	25	3	11	1
		SI	11.5	7.9	13.8	10
	Total infected mosquitoes		28		19	

N denotes the number of mosquitoes collected, N+ denotes the number of infected mosquitoes, and SI indicates the sporozoite index. The highest rates of infection were seen in Donia in both years.

One specimen of *An*. *funestus* was infected in Bongor in 2008 (SR = 6.2%). Infected *An*. *funestus* mosquitoes were found in Donia in both collection rounds in 2010 (SR round 1 = 7.9%, SR round 2 = 10%) but not in any other sentinel sites. Of the complementary vectors tested, low numbers of infected *An*. *pharoensis* were found in Bongor in the first collection round of 2010 and *An*. *nili* in Donia in the first collection round of 2010. No infected secondary vectors were found at the end of the rainy season.

## Discussion

In 2012, there were nearly 10 million people at risk of malaria in Chad and the country reported approximately 600,000 malaria cases [1]. Although ITNs and LLINs have been distributed free of charge since 2003 as the main vector control intervention, it is impossible to assess the success or otherwise of the programme, and to track trends in malaria incidence and insecticide resistance, without the collection of basic entomological data, including the composition and distribution of vector species and regular monitoring of their insecticide resistance status [1]. The WHO/TDR network for insecticide resistance in African malaria vectors was designed to elucidate trends in insecticide resistance and malaria vectors in five African countries and this report represents the first study of trends in vector composition and insecticide resistance from Chad.

Four sentinel sites with different selective pressures for insecticide resistance were selected and bioassays to detect insecticide resistance were performed biannually between 2008 and 2010. Following the routine monitoring period, additional collections were performed in selected sites in 2011 and 2014. During the routine monitoring period, the site with the greatest exposure to insecticide, Donia, showed the highest overall levels of insecticide resistance to DDT, deltamethrin and permethrin; a subsequent collection made in 2011 following the end of routine monitoring showed further decreases to mortality from these insecticides. Dramatic decreases in susceptibility to DDT and pyrethroid insecticides, after the end of the routine monitoring period, were detected in N'djamena (the capital city of the country) and Bongor. The first one is an urban agriculture site where pyrethroid insecticides are regularly applied for crops protection and for domestic use. In Bongor, a high coverage of insecticide-based malaria control interventions was implemented for several years. As the pyrethroid insecticides are the only class of insecticides currently approved for use in LLINs, Chad’s main malaria control intervention, these results require urgent investigation to determine the operational consequences of this resistance [20].

Both DDT and pyrethroid insecticides target the voltage gated sodium channel, and target site mutations (*kdr*) result in cross-resistance between compounds. *An*. *arabiensis* was predominantly found in N’Djamena, Mandelia and Bongor, whilst in Donia, *An*. *gambiae* M and S molecular forms were identified. *An*. *gambiae* S form was the only species to carry *kdr* alleles, with both 1014F and 1014S alleles being detected, in some cases in the same individual. The presence of the 1014F allele in *An*. *gambiae* S from Donia suggests that this allele may be widely distributed in the southern parts of Chad, in line with previous observations [9].

The absence of target site mutations in the *An*. *arabiensis* populations that were characterised indicates that a metabolic based mechanism may be present in this species in southern Chad. Testing performed with the synergist PBO, an inhibitor of cytochrome P450 enzymes, either fully or partially restored mortalities to deltamethrin and permethrin in mosquito populations from all four study sites; further evidence implicating P450-mediated resistance in *An*. *arabiensis* in Chad was found by biochemical analysis and genetic mapping studies on populations collected in N’Djamena [10]. As previous studies in this region have found extensive gene flow between *An*. *arabiensis* populations 300km apart [11], this suggests that this form of resistance could spread rapidly throughout the country. Metabolic based resistance is increasingly being observed in Africa [20] and is a concern as this type of resistance has been associated with malaria programme failure [21]. Understanding this resistance will allow for the implementation of an Insecticide Resistance Management Plan as outlined by GPIRM [22]

As expected, given that all collections were made during the rainy season, all adult Anopheline mosquito collections were comprised predominantly of *An*. *gambiae* s.l. [23,24]. However, consistently fewer *An*. *gambiae* were collected in the late rainy season compared to the early rainy season, which is unexpected given previous data from south-Western Chad that show *An*. *arabiensis* peaks in October and May [7]. Although *An*. *funestus* was collected in indoor catches in Bongor and Donia in 2009 and 2010, overall it was not abundant. This supports earlier findings in Chad that the breeding habitats for this species are becoming less frequent due to continuing local environmental modifications [7].

Of the Anopheline mosquitoes tested for *P*. *falciparum* circumsporozoite protein, the highest infection rate was seen in Donia (15.5% and 13.8% in 2008 and 2010, respectively, Table 4), the site with extensive agricultural use of pyrethroid insecticides and the highest levels of insecticide resistance (Fig 2). This is much higher than in a previous study performed in Chad [7] and in a comparable study performed in two Sahelian regions in Niger in 2007, which found sporozoite rates of 3.0% in *An*. *gambiae* s.l. in sites with crop cultivation but no malaria control interventions [25]. This could be partly explained by an overestimation of sporozoite rate using the ELISA method [26], but the rates seen in Donia are higher than this correction factor allows. Sporozoite prevalence can vary from location to location, however a recent study hypothesised that rates of mosquito infection are resilient to changes in endemicity, which could have implications for the further deployment of transmission-reducing interventions in Chad [27].

All of the sites in this study were located in the south of Chad, where the distribution of LLINs is the current malaria intervention strategy. Given that collections made after the end of the routine monitoring period showed rapid increases to pyrethroid resistance, there is a need to investigate whether the levels of insecticide resistance seen in the bioassays have translated to compromised efficacy, so that strategies to mitigate the effects of this resistance can be implemented [20,28]. One such strategy may be the use of PBO-treated LLINs, as the use of PBO synergised resistance in all four study sites, and these nets have showed increased efficacy against pyrethroid resistant populations compared to conventionally-treated LLINs [29].

During the monitoring gap of four years, significant changes occurred in resistance prevalence in 3 of the 4 sites (p = <0.001), endangering the efficacy of currently implemented malaria control interventions. Significant changes in insecticide resistance profiles and a lack of *kdr* resistance alleles in adult populations highlight the urgent need for comprehensive entomological monitoring to be implemented and maintained in country along with the development of an insecticide resistance management plan [22,30–32].

## Supporting Information

S1 Table Percentage mortality in bioassay tests. (PDF)

S2 Table *An*. *gambiae* species distribution and *kdr* genotyping results for all sites over three years of routine monitoring. (PDF)
