# Supplementary material for: Spatial and Temporal Trends in Insecticide Resistance among Malaria Vectors in Chad Highlight the Importance of Continual Monitoring
Source: PLoS One. 2016 May 26;11(5):e0155746. doi: 10.1371/journal.pone.0155746 (PMC4881902; doi:10.1371/journal.pone.0155746)
Supplement: S1 Table — (PDF) [file pone.0155746.s001.pdf]

Insecticide	Year	Collection	Locality			
			N'Djamena	Mandelia	Bongor	Donia
**DDT**	2008	1	100	100	96	24
	2008	2	97	100	89	86
	2009	3	94.6	97.3	84.2	19.6
	2009	4	90.4	87.4		11.1
	2010	5	98.2	100	100	22.9
	2010	6	94.5	95	98	82
	2011	7				32
	2014	8	0		24	
**Deltamethrin**	2008	1	64	93	80	73
	2008	2	100	74	31	68
	2009	3	73.9	82.8	53.7	55.8
	2009	4	87	95.6		82
	2010	5	84	86.7	83	48.5
	2010	6	70.4	76.5	85.8	47
	2011	7				36
	2014	8	2		35	
**PBO/ Deltamethrin**	2008	1				
	2008	2				
	2009	3		100	100	
	2009	4	100	100		97.4
	2010	5				
	2010	6		100	100	
	2011	7				
	2014	8				
**Permethrin**	2008	1	70	93	74	65
	2008	2	72	50.3	43	75
	2009	3	49.1	94.1	63.7	55.3
	2009	4	74.5	56.9		89.7
	2010	5	94.1	83.7	90.6	43.3
	2010	6	72.6	89.7	89	62.2
	2011	7				38
	2014	8	2		36	
**PBO/ Permethrin**	2008	1				
	2008	2				
	2009	3		100	100	
	2009	4	100	100		
	2010	5				
	2010	6			100	
	2011	7				
	2014	8				
**Bendiocarb**	2008	1	100	100	100	100
	2008	2	100	100	100	100
	2009	3	100	100	100	100
	2009	4	100	100		100
	2010	5	100	100	100	100
	2010	6	98.9	100	100	100
	2011	7				100
	2014	8	100		100	

Insecticide	Year	Collection	Locality			
			**N'Djamena**	**Mandelia**	**Bongor**	**Donia**
**Fenitrothion**	2008	1	100	100	100	100
	2008	2	100	100	100	100
	2009	3	100	100	100	100
	2009	4	100	100		100
	2010	5	100	100	100	100
	2010	6	100	100	100	100
	2011	7				100
	2014	8	100		100	
