# Supplementary material for: Spatial and Temporal Trends in Insecticide Resistance among Malaria Vectors in Chad Highlight the Importance of Continual Monitoring
Source: PLoS One. 2016 May 26;11(5):e0155746. doi: 10.1371/journal.pone.0155746 (PMC4881902; doi:10.1371/journal.pone.0155746)
Supplement: S2 Table — (PDF) [file pone.0155746.s002.pdf]

Collections	Localities	Species	N	Genotype at the *kdr* locus						f (Ser)	f (Phe)
				Leu-Leu	Leu-Phe	Phe-Phe	Leu-Ser	Ser-Ser	Phe-Ser		
**Round 1**	N'Djaména	*An. arabiensis*	40	40	0	0	0	0	0	0	0
	Mandélia	*An. arabiensis*	39	39	0	0	0	0	0	0	0
	Bongor	*An. arabiensis*	37	37	0	0	0	0	0	0	0
		An. gambiae M	3	3	0	0	0	0	0	0	0
	Donia	*An. arabiensis*	3	3	0	0	0	0	0	0	0
		An. gambiae M	14	14	0	0	0	0	0	0	0
		An. gambiae S	19	1	4	3	4	3	4	0.37	0.37
**Round 2**	N'Djaména	*An. arabiensis*	37	37	0	0	0	0	0	0	0
	Mandélia	*An. arabiensis*	39	39	0	0	0	0	0	0	0
	Bongor	*An. arabiensis*	40	40	0	0	0	0	0	0	0
	Donia	*An. arabiensis*	9	9	0	0	0	0	0	0	0
		An. gambiae M	17	17	0	0	0	0	0	0	0
		An. gambiae S	12	6	3	0	3	3	3	0.13	0.13
**Round 3**	Donia	*An arabiensis*	1								
		An gambiae M	7								
		An gambiae S	32								
**Round 4**	Donia	*An arabiensis*	27								
		An gambiae M	7								
		An gambiae S	6								
**Round 5**	N'Djemena	*An arabiensis*	40	40	0	0	0	0	0	0	0
	Mandelia	*An. arabiensis*	40	40	0	0	0	0	0	0	0
	Bongor	*An. arabiensis*	40	40	0	0	0	0	0	0	0
**Round 6**	N'Djaména	*An. arabiensis*	39	39	0	0	0	0	0	0	0
		An. gambiae M	1	1	0	0	0	0	0	0	0
	Mandelia	*An arabiensis*	40	40	0	0	0	0	0	0	0
	Bongor	*An arabiensis*	40	40	0	0	0	0	0	0	0
		Total	629								
